# Direct measurement on the geometric phase of a double quantum dot qubit via quantum point contact device

**DOI:** 10.1038/srep11726

**Published:** 2015-06-29

**Authors:** Bao Liu, Feng-Yang Zhang, Jie Song, He-Shan Song

**Affiliations:** 1 Beijing Computational Science Research Center (CSRC), Beijing 100094, China; 2 School of Physics and Materials Engineering, Dalian Nationalities University, Dalian 116600, China; 3 Department of Physics, Harbin Institute of Technology, Harbin, 150001, PR China; 4 School of Physics and Optoelectronic Technology, Dalian University of Technology, Dalian 116024, China

## Abstract

We propose a direct measurement scheme to read out the geometric phase of a coupled double quantum dot system via a quantum point contact(QPC) device. An effective expression of the geometric phase has been derived, which relates the geometric phase of the double quantum dot qubit to the current through QPC device. All the parameters in our expression are measurable or tunable in experiment. Moreover, since the measurement process affects the state of the qubit slightly, the geometric phase can be protected. The feasibility of the scheme has been analyzed. Further, as an example, we simulate the geometrical phase of a qubit when the QPC device is replaced by a single electron transistor(SET).

In recent years, quantum computation and quantum information are developing rapidly. As a result people have devoted much effort in searching for physical settings as quantum bits, such as quantum optical system^1^,^2^, diamond NV center^3^,^4^,^5^ and quantum dot system^6^,^7^,^8^. Quantum dot is a promising candidate of solid-state qubit. The number of charges, which confined in a quantum dot can be controlled by electrical gates surrounding it. Quantum dot has the merit that the charges and spins confined in it can be directly manipulated optically or electrically, and has a long coherent time.

The fluctuation of charges or nuclear spins will diminish the coherence time of quantum dot qubits^9^,^10^. Combating decoherence is an critical task in quantum memory. Geometric phase, which is robust to the fluctuation of the bath, is an important resource to construct phase gates^9^,^11^ in quantum information systems.

Theoretically geometric phase was discovered in context of adiabatic and cyclic closed quantum system by Berry in 1984^12^, and then it has been generalized to non-adiabatic cyclic system, non-adiabatic and non-cyclic system^13^,^14^,^15^,^16^,^17^,^18^,^19^,^20^,^21^. Recently, Yin and Tong have studied the effect of the environment on the geometric phase in open quantum dot qubit system^22^,^23^. However, the methods to get the geometric phase are mainly by interference effect of the system. As the method proposed by Pancharatnam, in a quantum optical system, people usually need to compare phases of two beams of polarized light. The measurement of the geometric phase always lead to the destruction of information carried by the quantum system. Therefore, to propose a scheme that the geometric phase can be measured without spoiling information embedded in quantum system is very important and interesting for the fundamental concepts of quantum theory and the quantum information process.

In this paper by studying a well known model we propose a direct measurement scheme on the geometric phase via the current through the QPC device. Since the QPC affects the quantum state of the double quantum dot system slightly^24^,^25^, the read out operation conserves the phase information. Then we studied the feasibility of the scheme. As an example of our theory we simulate the geometric phase when the QPC device is replaced by a SET.

## Results

### Model and master equation

**Figure 1:**
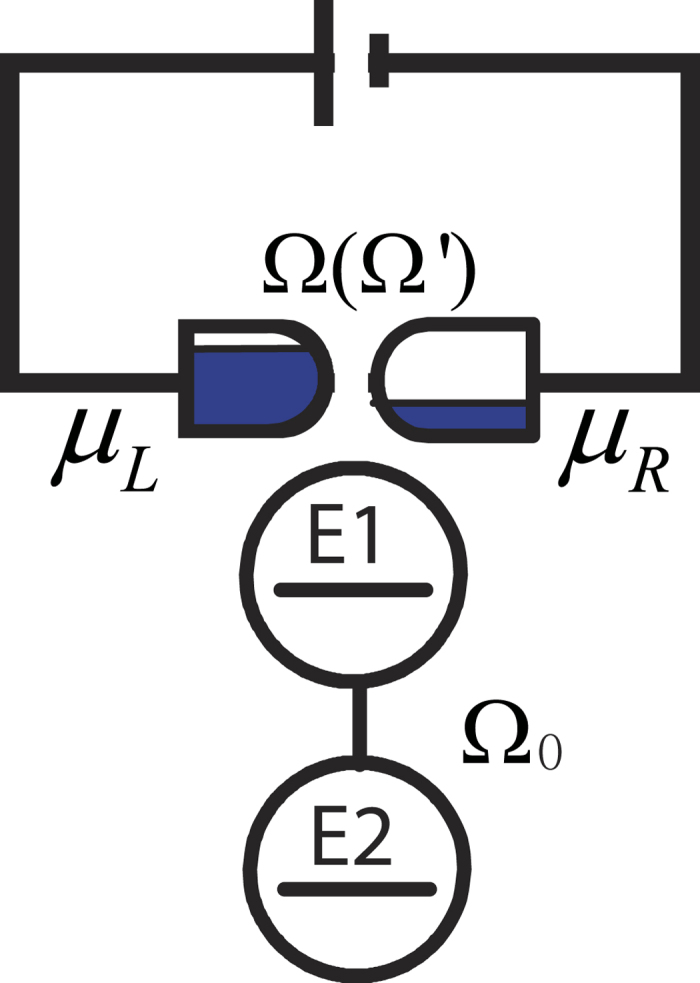
A typical model for quantum point contact measurement. In this model the energy level of the two quantum dot are *E*_1_ and *E*_2_ respectively. Ω_0_ is the coupling coefficient of the qubit. The potential between electrodes varies dependent on the place of the electron on the qubit.

Our model composed by a double quantum dot qubit and a QPC device as shown in Fig. 1. Two quantum dots in the qubit are coupled to each other with the strength Ω_0_. We assume that there is only one energy level in each quantum dot(*E*_1_ and *E*_2_). One electron is confined in the qubit and tunnels between these two levels. The QPC device contains two leads. The chemical potential of the left lead *μ*_*L*_ is higher than that of the right lead *μ*_*R*_. Therefore, electrons can tunnel from left lead to right lead. The qubit interacts with the QPC device by changing the coupling strength between two leads. When the electron in the qubit occupies *E*_2_ state, the coupling strength between two leads is Ω. Once the electron jumps to *E*_1_ state, the coupling strength will be changed to Ω′. At low temperature two leads of the QPC are filled to their Fermi energies by electrons. The Hamiltonian of our model reads


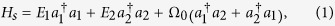



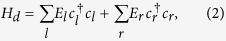



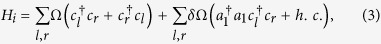


where *a*_*i*_ and 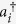
(*i* = 1, 2) are the annihilation and creation operators of the electron confined in the qubit. While *c*_*l*/*r*_ and 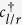 are the annihilation and creation operators of the electrons in the left/right lead, respectively. Here *E*_*l*/*r*_ is the energy of left/right lead. We defined *δ*Ω = Ω − Ω′. *H*_*s*_, *H*_*d*_ are the Hamiltonian of the qubit and the QPC device, respectively. The QPC is connected to a large electron source. Therefore, the Fermi energy of each lead is not changed by the tunneling between two leads. And the voltage *V*_*d*_ between two leads is a constant. *H*_*i*_ describes the interaction between the qubit and the QPC device. Hence, there is no electron tunnels between leads and the qubit.

Since the the whole system contains qubit and QPC device is a closed system. From the Schödinger equation of the whole system 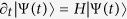, and the method proposed by Gurvitz^26^,^27^,^28^,^29^, we obtain a hierarchical equation of the qubit


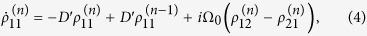



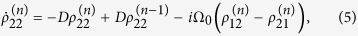



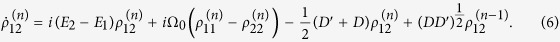


In these expressions *ρ* is the reduced density matrix of the qubit. The bases of *ρ* are |1〉 and |2〉, where |*i*〉 means the electron in the qubit occupies *E*_*i*_(*i* = 1, 2) energy level. Hence, the superscription *n* counts the number of electrons, which passed the QPC^30^,^31^. Here *D* = 2*π*|Ω|^2^*ρ*_*l*_*ρ*_*r*_*V*_*d*_(*D*′ = 2*π*|Ω′|^2^*ρ*_*l*_*ρ*_*r*_*V*_*d*_) is the coupling strength between the two leads when the electron in the qubit occupies *E*_2_(*E*_1_) energy level. We have used *ρ*_*l*/*r*_ to describe the density of states of the left/right lead^32^,^33^.

Sum of the superscriptions of the hierarchical equation we obtain a master equation of the qubit as


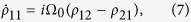



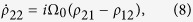



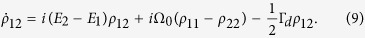


This master equation can be abbreviated to


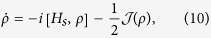


where 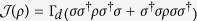 is the dissipative part. We have defined 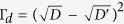 as the decoherence rate of the qubit. Hence, *σ* = |*E*_1_〉〈*E*_2_| is the pseudospin operator of the qubit.

Further, we can obtain the current through the QPC device as


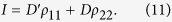


### Direct measurement scheme on geometric phase

We use the formula proposed by Tong *et al.* in Refs. 18,19 to calculate the geometric phase of the qubit. The geometric phase of a two level system can be written as


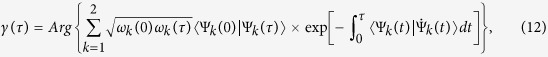


where *ω*_*k*_(0), *ω*_*k*_(*τ*) and |Ψ_*k*_(0)〉, |Ψ_*k*_(*τ*)〉(*k* = 1, 2) are the instantaneous eigenvalues and eigenvectors of the qubit at time *t* = 0, *τ*. For simplicity the initial condition of the qubit is taken as *ρ*_11_ = 1 and *ρ*_22_ = *ρ*_12_ = *ρ*_21_ = 0. Under this initial condition the geometric phase of the qubit is


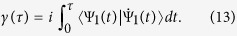


An arbitrary density matrix of a two level system can maps in a Bloch sphere


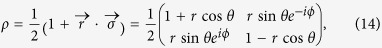


where *r* is the length of the Bloch vector. We have defined *θ*(*zenith angle*) and *ϕ*(*azimuth angle*) to depict the direction of the Bloch vector. Without lose of generality, we obtain the instantaneous eigenvalues and the eigenvactors of an arbitrary two level system as


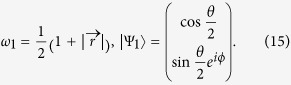



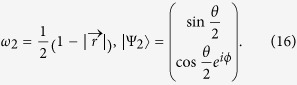


The initial condition of the qubit implies that *ω*_1_(0) = 1, *ω*_2_(0) = 0 and 
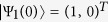
, 
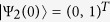
. Therefore, the geometric phase of our system reads

The initial condition of the qubit implies that *ω*_1_(0) = 1, *ω*_2_(0) = 0 and 
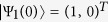
, 
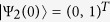
. Therefore, the geometric phase of our system reads


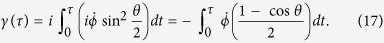


Finally, with our method we obtain a simple expression of the geometrical phase, which relates the geometric phase of the qubit to the current through the QPC device.


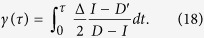


Here Δ = *E*_2_ − *E*_1_ is the energy difference between the two levels in the qubit.

It has a very important merit that all the parameters in the expression are observable and can be measured or tuned in experiment. From the expression of the current Eq. (11) we find that *D*(*D*′) is nothing but the current through the QPC when the electron in the qubit occupies *E*_2_(*E*_1_) energy level. We can trap the electron in the qubit in *E*_2_(*E*_1_) by the gate electrode between these two dots, while the current through the QPC device is *D*(*D*′). Hence, Δ can be tuned by the back electrodes behind the two quantum dots. The formula Eq. (18) is feasible when the qubit is weakly measured. Moreover, since the QPC device does not damage the state of the qubit, our measurement scheme can protect the information of geometric phase against destruction.

**Figure 2:**
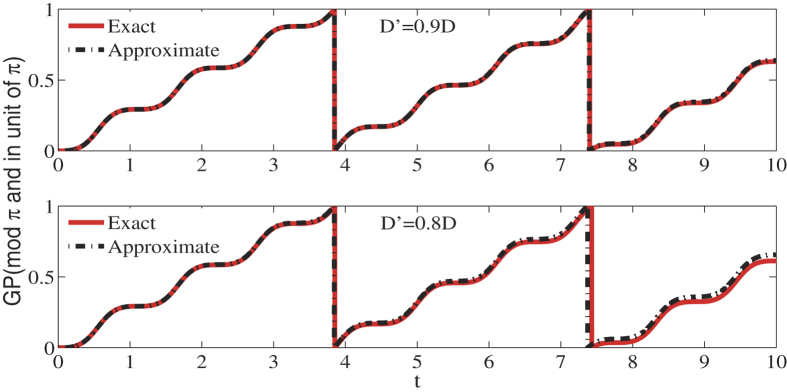
In these figures we compare the geometric phases from Eq. (18)(dash-dot line) and the exact ones(red solid line). The parameters are chosen as Ω_0_ = 2, Δ = 4 and *D* = 1. In upper and lower figure *D*′ = 0.9*D* and *D*′ = 0.8*D*, respectively.

In Fig. 2 we show the geometric phases of the qubit(red solid line) and the results from Eq. (18)(dash-dot line). In these two figures we take modulus of the geometrical phase *γ*(*τ*) by *π* and take *π* as a unit. The parameters are chosen as Ω_0_ = 2, Δ = 4 and *D* = 1. In the upper figure the geometric phase from Eq. (18) matches the exact solution well. The lower figure shows, the geometric phase from our formula differs gradually from the actual one as *D*′ decreases.

### The feasibility of the measurement scheme

In this section we proceed to analyze the feasibility of our direct measurement scheme. There are two major factors affecting the measurement result of the scheme. One is the length change of Bloch vector. If it varies too fast the approximation we used will be invalid. The other one is the current quality measured by the QPC device.

We first analyze the influence of the length change of the Bloch vector. We rewrite Eq. (31) in spherical polar coordinate as


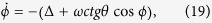



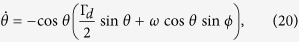



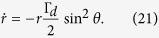


Since the differential equation of *r* is decoupled from the other two equations. The solution of this equation is


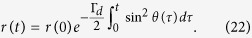


In Eq. (18) we have assumed that the length change of the Bloch vector is slow enough. The slower the length change of Bloch vector the more accurate Eq. (18) will be. According to Eq. (22), the length change of the Bloch vector is affected by Γ_*d*_ and *θ*. A small Γ_*d*_ can be obtained via diminishing the difference of the distances between the QPC and two quantum dots of the qubit. The second factor affecting the length change of Bloch vector is the path trace of the qubit in Bloch spere. During the evolution, the smaller *θ* is the more slowly *r* diminishes. To keep *θ* small, we need a properly large Δ. Therefor the path trace of the qubit in Bloch sphere approaches the north pole.

The other factor to affect the feasibility of Eq. (18) is the quality of the QPC current. According to Ref. 34 the Signal-to-Noise ratio of our model can be expressed as 
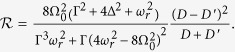
 Where 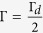, 
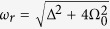
 To obtain a high quality measurement current we need a smaller difference between *D* and *D*′. This result is consistent with the analysis above.

### An application of the direct measurement scheme

Recently, Yin and Tong have studied the effects of environment parameters on the geometric phase of quantum dot systems^22^,^23^. Moreover, in Ref. 23 a model similar to ours is studied, in which the QPC device is replaced by a SET. In this section, as an application of our scheme we investigate how to simulate the time evolution of the geometric phase in this model via Eq. (18). Further, We provide a method to determine the parameters, with which we can obtain the geometric phase from our model.

In case that there is no backaction^23^. With definitions 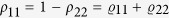 and 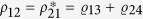 the master equation can be simplified as


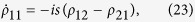



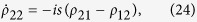



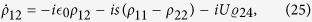



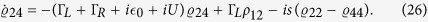


For simplicity, here we choose a condition that the coupling strength of the left lead to *QD*_0_ is smaller than the strength of *DQ*_0_ to right lead(Γ_*L*_ = 1, Γ_*R*_ = 8). Under this condition *QD*_0_ have a very small probability to be occupied. Therefore, 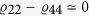 and the variation of 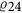 is very little, namely, 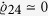. Hence, we can adiabatically replace 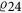 in Eq. (25) by 
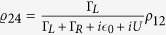
. Then we obtain an effective equation of motion as


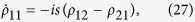



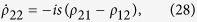



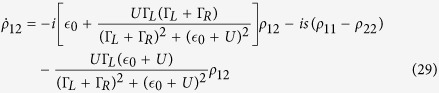


Obviously, this master equation have a similar form as Eq. (7, 8, 9). Hence, these two master equations have the same steady state. Therefore, we can simulate the time evolution of geometric phase with Eq. (18). Comparing these two master equations we obtain parameters Ω_0_ = −*s* 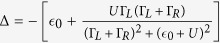 and 
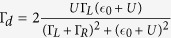
, with which we can obtain the geometric phase from our setup.

**Figure 3:**
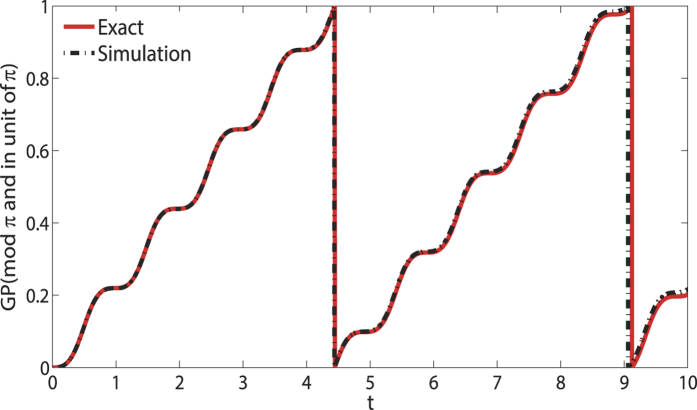
In this figure we simulate the time evolution of geometric phase of Yin and Tong’s model(red solid line) with Eq. (18)(dash-dot line). The parameters are chosen as *s* = 2, 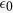 = 5, Γ_*L*_ = 1, Γ_*R*_ =  8 and *U* = 0.1.

In Fig. 3 we compare the exact geometric phase from the model of Ref. 23 (red solid line) and the result from Eq. (18) (dash-dot line). In this figure the parameters are chosen as *s* = 2, 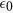 = 5, Γ_*L*_ = 1, Γ_*R*_ = 8 and *U* = 0.1.

## Discussion

In conclusion, we have proposed a direct measurement scheme on the geometric phase of a double quantum dot qubit via the QPC current. An effective formula, which relates the geometric phase to the QPC current has been derived. All parameters in our expression are measurable in experiment. Moreover, since the QPC device affects the state of the qubit slightly, our measurement procession protects the geometric phase from destruction. The feasibility of the scheme has been studied. When the QPC measurement is weak, the measurement scheme will be feasible. As an application of our theory, we simulate the evolution of the geometric phase of the model in Ref. 23, in which the QPC is replaced by a SET. This simulation shows the usefulness of our scheme. This investigation should be helpful to design experiment setups based on quantum dot systems, which can measure the information of geometric phase without damaging the phase.

## Methods

For Eq. (18), we map master equation Eq. (10) onto the Bloch sphere. Under the Cartesian coordinate system the new master equation in this space is


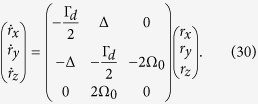


Here we define *r*_*x*_ = *ρ*_12_ + *ρ*_21_, *r*_*y*_ = *i*(*ρ*_12_ − *ρ*_21_), *r*_*z*_ = *ρ*_11_ − *ρ*_22_, *ω* = 2Ω_0_ and Δ = *E*_2_ − *E*_1_. Under spherical polar coordinate system we have *r*_*x*_ = *r* cos*θ* cos*ϕ*, *r*_*y*_ = *r* sin*θ* sin*ϕ* and *r*_*z*_ = *r* cos*θ*. Master equation Eq. (30) can be rewritten as


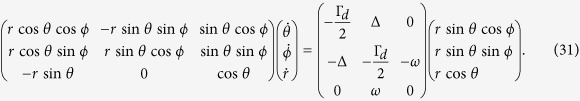


If the decoherence rate Γ_*d*_ is small. In a short time scale, the path trace of Eq. (30) approximately parallel to the path trace of a close system, where Γ_*d*_ = 0. We first study the dynamics of this close system. Under initial condition *ρ*_11_ = 1 and *ρ*_22_ = *ρ*_12_ = *ρ*_21_ = 0, the solution of the closed system reads


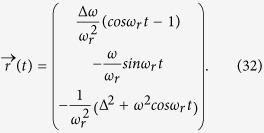


Here 
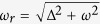
 is the Rabi frequency of the qubit. The path trace in Bloch sphere when 
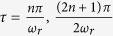
 passes two point on the *xoz* plane


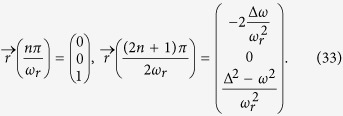


Further, since the orbit is perpendicular to the *yoz* plane, we can parameterize the projection of the orbit on *xoz* coordinate plane
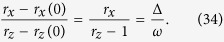
According to the initial condition we have
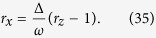


If the length of the Bloch vector 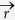 changes slowly. In a short time scale we can omit the length change of the Bloch vector, namely, 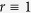. With (31) and (35) we obtain an effective formula of the azimuth angle


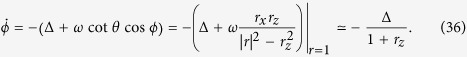


Further, in a short time scale we have 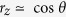
(*r* = 1). Finally by using all these approximations we obtain an expression of the geometrical phase as
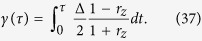
According to the expression of current Eq. (11), *r*_*z*_ can be rewritten as


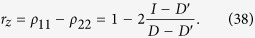


Insert this expression into Eq. (37), we obtain formula Eq. (18).

## Additional Information

**How to cite this article**: Liu, B. *et al.* Direct measurement on the geometric phase of a double quantum dot qubit via quantum point contact device. *Sci. Rep.* **5**, 11726; doi: 10.1038/srep11726 (2015).
